# Doxorubicin causes cachexia, sarcopenia, and frailty characteristics in mice

**DOI:** 10.1371/journal.pone.0301379

**Published:** 2024-04-22

**Authors:** Paola Sanches Cella, Ricardo Luís Nascimento de Matos, Poliana Camila Marinello, Júlio Cesar da Costa, Felipe Arruda Moura, Ana Paula Frederico Rodrigues Loureiro Bracarense, Patricia Chimin, Rafael Deminice

**Affiliations:** 1 Department of Physical Education, State University of Londrina, Londrina, Paraná, Brazil; 2 Laboratory of Chemistry, State University of Londrina, Londrina, Paraná, Brazil; 3 The University of Texas MD Anderson Cancer Center, Houston, Texas, United States of America; 4 Laboratory of Applied Biomechanics State University of Londrina, Londrina, Paraná, Brazil; 5 Laboratory of Animal Pathology, State University of Londrina, Londrina, Paraná, Brazil; Bursa Ali Osman Sonmez Oncology Hospital, TURKEY

## Abstract

While chemotherapy treatment can be lifesaving, it also has adverse effects that negatively impact the quality of life. To investigate the effects of doxorubicin chemotherapy on body weight loss, strength and muscle mass loss, and physical function impairments, all key markers of cachexia, sarcopenia, and frailty. Seventeen C57/BL/6 mice were allocated into groups. 1) Control (n = 7): mice were exposed to intraperitoneal (i.p.) injections of saline solution. 2) Dox (n = 10): mice were exposed to doxorubicin chemotherapy cycles (total dose of 18 mg/kg divided over 15 days). The body weight loss and decreased food intake were monitored to assess cachexia. To assess sarcopenia, we measured muscle strength loss using a traction method and evaluated muscle atrophy through histology of the gastrocnemius muscle. To evaluate physical function impairments and assess frailty, we employed the open field test to measure exploratory capacity. Doxorubicin administration led to the development of cachexia, as evidenced by a significant body weight loss (13%) and a substantial decrease in food intake (34%) over a 15-day period. Furthermore, 90% of the mice treated with doxorubicin exhibited sarcopenia, characterized by a 20% reduction in traction strength (p<0,05), a 10% decrease in muscle mass, and a 33% reduction in locomotor activity. Importantly, all mice subjected to doxorubicin treatment were considered frail based on the evaluation of their overall condition and functional impairments. The proposed model holds significant characteristics of human chemotherapy treatment and can be useful to understand the intricate relationship between chemotherapy, cachexia, sarcopenia, and frailty.

## 1 Introduction

Cachexia, sarcopenia, and frailty are prevalent conditions in cancer patients and are strongly linked to unfavorable outcomes, including poor cancer prognosis, extended hospital stays, heightened treatment expenses, increased risk of falls, and elevated mortality rates [1, 2]. Cachexia alone accounts for approximately 20% of all cancer-related deaths and serves as an indicator of poor prognosis [1]. Similarly, sarcopenia has been detected in 35% of cancer patients and can achieve >50% in some types of cancer such as oesophageal cancer, urothelial cancer, cholangiocarcinoma, sarcomas, and thyroid cancer [3]. Markedly, sarcopenia is associated with heightened surgical complications, increased chemotherapy toxicity, and reduced survival rates in various malignancies such as breast, colorectal, lung, kidney, ovarian, and lymphoma [3–5]. Although there is comparatively less research on cancer related-frailty, a meta-analysis involving 2,916 participants with different stages of solid or hematological malignancies revealed that over half of older cancer patients exhibited pre-frailty or frailty, and these individuals faced an elevated risk of chemotherapy intolerance, postoperative complications, and mortality [2].

Although chemotherapy treatment is a lifesaving intervention for several types of cancer, it is notable that it can further exacerbate cancer-related cachexia, sarcopenia, and frailty [6]. A prospective study conducted by Daly et al. [6], involving adult foregut cancer patients, demonstrated that chemotherapy led to a significant loss of muscle mass. Specifically, the study demonstrated that chemotherapy resulted in a loss of 6.1 cm^2^ of skeletal muscle per 100 days, which corresponds to a loss of 1.0 kg of skeletal muscle mass and 2.0 kg of fat-free mass when considering the entire body. They concluded that a substantial reduction in muscle mass is predictive of decreased survival in patients undergoing palliative chemotherapy. Furthermore, frailty can increase from 13% to 43% following chemotherapy treatment [7]. Taken together, these data are relevant given that cachexia, sarcopenia, and frailty may negatively impact the quality of life and treatment outcomes for cancer patients [8–12].

Cachexia, sarcopenia, and frailty play crucial roles in cancer development and prognosis, there is currently a lack of approved drugs specifically designed for their treatment. Consequently, pre-clinical models investigating muscle and functional decline induced by chemotherapy have become vital for understanding the mechanisms underlying these conditions. Additionally, these models serve as a platform for exploring therapeutic strategies to mitigate the detrimental changes associated with cachexia, sarcopenia, and frailty. Through pre-clinical research, potential interventions and treatment options can be investigated to address the challenges posed by these conditions. For this purpose, doxorubicin was chosen for this study due to its widespread use as a first-line chemotherapy treatment in advanced stages of various types of cancer, and its known association with several adverse effects, including myopathies [13].

Therefore, our objective was to examine whether chemotherapy treatment with doxorubicin induces weight and muscle strength loss, muscle wasting, and physical impairments, all of which are key indicators of cachexia, sarcopenia, and frailty. We hypothesized that doxorubicin-based chemotherapy treatment would lead to weight loss, muscle strength decline, muscle wasting, and physical impairments, making it a valuable preclinical model to study these parameters.

## 2 Material and methods

### Animals and treatment

C57/BL/6 male mice (~90 days old) were obtained from the facilities of the State University of Londrina Animal Care Unit. Mice were kept in collective cages (~4 mice/cage) with full access to food (Nuvilab^®^ balanced CHO pellets, Nuvital SA, Colombo, Brazil) and water, at controlled room temperature (26 ± 1°C). According to previous studies conducted in our laboratory [14, 15], a total of seventeen mice were randomly assigned into one of two groups designated as Control (C, n = 7) and treated with doxorubicin (Dox, n = 10). The Dox group had a larger sample size due to the possibility of animal mortality. If the animal had a weight loss of more than 20% in less than a week, it was euthanized. Additionally, severe impairments such as impaired walking, frequent diarrhea, accelerated breathing, bristly and dull hair, also warranted euthanization.

The randomization sequence was generated by the researcher through an aleatory (random) selection process. We implemented a systematic approach to minimize potential confounders in our experimental design. The address the issue of treatment and measurement order, we randomized the sequence in which treatments were administered and measurements were taken. This randomization was performed for each experimental group to ensure that any potential bias introduced by the order of events was evenly distributed across all groups. Regarding the potential influence of cage location, we employed a controlled environment where animals were housed in identical cages with consistent environmental conditions.

To replicate the chemotherapy treatment used in humans, we administered doxorubicin hydrochloride (Bergamo, Brazil) in cycles, with a dosage of 6mg/kg every five days for a total of two weeks (resulting in a total dosage of 18 mg/kg). The drug was injected intraperitoneally, while the control group received an equivalent volume of PBS as a vehicle. We monitored the mice’s body weight and food intake daily (Shimadzu do Brasil Comércio scale LTDA-SP). All mice were euthanized at the end of the 15^th^ experiment day. All procedures and protocols implemented in this study were conducted in accordance with the guidelines set by the National Institutes of Health- guide for the care and use of laboratory animals. Experimentation (CONCEA) and were approved by the Ethical Committee for Animal Research (CEUA) of Londrina State University, Brazil (Protocol CEUA #11131.2019.07).

### Euthanasia and tissue preparation

After two weeks of doxorubicin treatment, mice were anesthetized using 5% isoflurane (Instituto Biochimico IND. Farm. LTDA- RJ) and euthanized by cervical dislocation. The gastrocnemius skeletal muscle was dissected, weighed, and stored for histological analysis, as described below.

### Body weight and food intake measurements for cachexia determination

Cachexia was based on the criteria described by Bozzetti and Mariani [16], which involve the combination of changes in body weight and food intake. Mice were classified as pre-cachexia if they had a body weight loss of up to 10% accompanied by a reduced food intake. Cachexia was diagnosed when the mice exhibited a body weight loss exceeding 10% along with reduced food intake. Reduced food intake was considered when food consumption of the doxorubicin group was statistically significantly lower (p< 0,05) than control. Total body weight and food intake were determined daily during all the experimental periods.

### Muscle strength and mass loss for sarcopenia determination

Sarcopenia was evaluated using the k-means cluster method. Each of the three key indicators of sarcopenia (muscle strength, muscle mass, and physical performance) was classified into two categories: normal and low. The classification system was adapted from the consensus guidelines of the European Working Group on Sarcopenia in Older People 2 (EWGSOP2) for humans and applied to mice, resulting in four distinct categories: 1) Mice that did not exhibit low strength–even if they had low muscle mass and/or low physical function, they were classified as non-sarcopenic. 2) Mice displaying low muscle strength alone were classified as pre-sarcopenic. 3) Those demonstrating both low muscle strength and low muscle mass were categorized as sarcopenic. 4) Mice with low muscle strength, low muscle mass, and low physical function were classified as severe sarcopenia.

Strength was assessed using a grip strength meter (Insight, Brazil). Mice underwent the test by gripping a traction bar with their upper limbs while being gently pulled by their tail. The precision force gauges on the grip strength meter measured the peak force applied and displayed the value digitally. A traction force sensor in the grip strength meter recorded the peak force exerted by the mice. The researcher recorded this measurement [17]. Each mouse was given five attempts, and the average of these attempts was used for analysis.

The muscle mass analysis was determined using the cross-sectional area (CSA) of the skeletal muscle fibers in μm^2^, assessed by optical microscopy. The gastrocnemius muscle was fixed in 4% formaldehyde for 24 hours, followed by dehydration with graded ethanol, and embedded in paraffin blocks, following the protocol described by Fonseca et al. [18]. The gastrocnemius muscle was cut into sections with a thickness of 5 μm. Two sections per mouse were prepared. These sections were stained with hematoxylin and eosin (H&E), and five images were captured from each section at a magnification of 200x. To quantify the CSA, the area of approximately 15 fibers was measured from each image using the Image J program. This resulted in a total of ~750 fibers per group for quantification.

Exploratory activity was assessed (cm) as parameters of physical function using the open field test, following the methodology described by Voltarelli *et al*., [17]. For the test, an open field arena measuring 60 × 60 cm was used. Each mouse was placed in the center of the field arena and allowed to freely explore it for 30 seconds, followed by a 5-minute test duration. The overall exploratory activity was recorded using a video track digital camera (Logitech, C920) fixed above a rigid box. The recording was conducted at a frequency of 30 Hz, and the obtained video footage was analyzed using automatic tracking methods through the DeepLabCut software interface [19, 20].

In order to support the measurement of sarcopenia and explore alternative methods for future studies, additional approaches for assessing strength and muscle mass were also applied. Muscle strength was evaluated using the climbing test, and muscle mass was determined using the sum of muscles. Results demonstrated 90% consistency in determining sarcopenia using different strength and muscle mass methods (S1 Fig).

### Clinical frailty index

Frailty was determined using the index proposed by Whitehead et al. [21]. The index was calculated based on eight items reflecting locomotion, exploratory activity, and body weight loss. The locomotion and exploratory activity were measured using the open field test, as described earlier. The results of this test were then used to determine seven parameters: a) total distance moved in 5 minutes (cm); b) maximal distance moved between bouts of inactivity (cm); c) total duration of movement (seconds); d) percent of total time spent moving; e) the change in direction per distance unit moved, called meander (degrees/cm; from 0º to 180º); f) the average velocity of movement over 5 minutes (cm/s); and g) rearing frequency (number of occurrences/min). The total body weight measured 24 hours before euthanasia (the same day the open field test was performed) was also used in the frailty score.

The frailty score was calculated by comparing each mouse from the Dox group to the control group, using the median and standard deviation (SD) values obtained from the control group as a reference. To determine the severity of frailty, a scale from Whitehead et al. [21] was utilized. Mice that scored 0 were classified as having no deficit, those with a score of 0.5 were classified as having a mild deficit, and those with a score of 1 were classified as having a severe deficit.

### Statical analysis

To check if our data adhered to the assumptions of the selected statistical approach, we employed the Shapiro-Wilk test for normality. For parametric data such as body weight loss, food intake, muscle strength, frailty score and Σ muscle, we presented them as mean and standard deviations by independent t-test. For non-parametric results (cross-section area), we presented data as median (Md) and percentiles, and we used the Mann-Whitney test was used. For analyzing muscle strength progression, a two-way ANOVA followed by Dunn’s post-hoc test was conducted, with a significance level set at 5%. The software utilized for these statistical analyses was GraphPad Prism v.8.0.

## 3 Results

### Doxorubicin treatment reduces body weight and food intake, the hallmarks of cachexia

**Fig 1:**
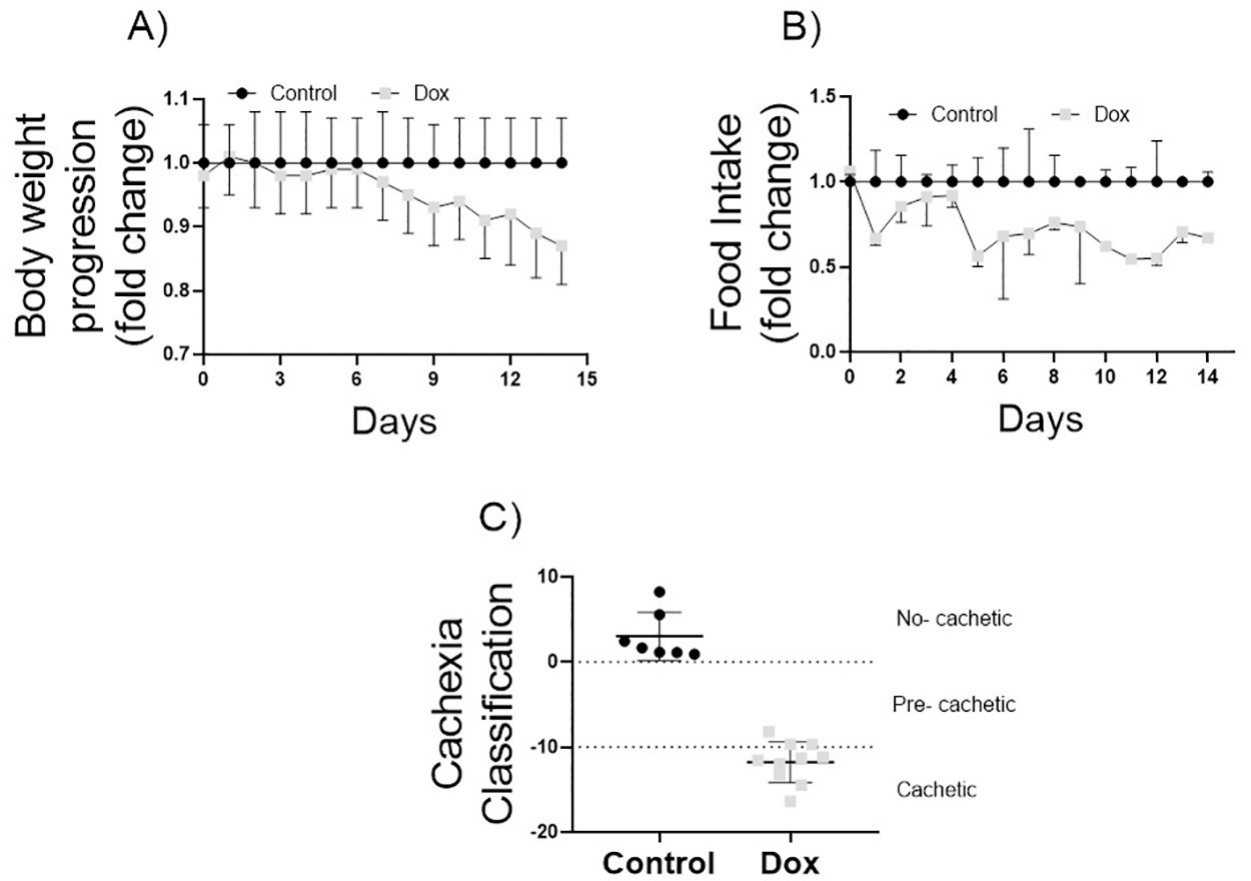
Hallmarks of cachexia and cachexia classification. A) Total body weight progression and B) food intake over the course of 15-day experiment, and C) cachexia classification for control group receiving saline injections (n = 7) and doxorubicin-treated groups (Dox, n = 10) with 6mg/kg every four days (a total dosage of 18 mg/kg). *Significant difference (P<0.05) from control group by ANOVA two-way for two independent sample in panel A and B, and by student-t test in panel C.

Fifteen days of doxorubicin treatment caused a significant (P<0.05) decrease in total body weight from day 11 to 15, representing a total loss of 13% (Fig 1A). The administration of doxorubicin resulted in a significant reduction in food intake starting from day 11 and continuing until the end of the experiment. This decrease in feeding amounted to a 34% reduction on the last day of the experiment when compared to the control group (Fig 1B). All animals treated with doxorubicin achieved some degree of cachexia; 30% presented pre-cachexia and 70% were classified as cachectic (Fig 1C).

### Doxorubicin promotes strength loss and muscle atrophy, the hallmarks of sarcopenia

**Fig 2:**
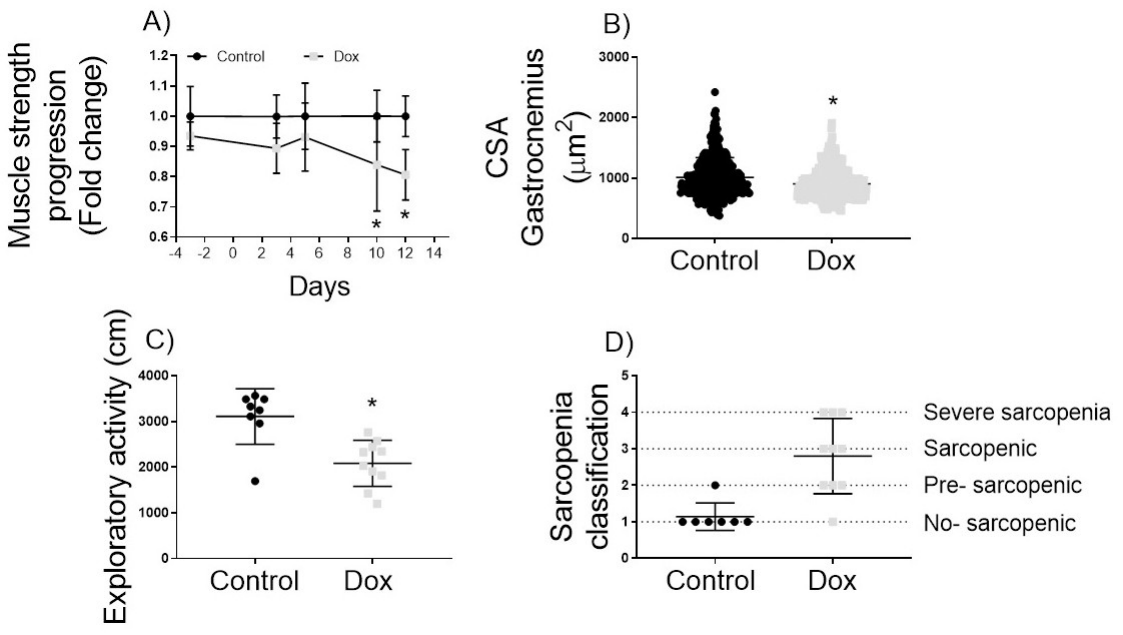
Hallmarks of sarcopenia and sarcopenia classification. A) Static muscle strength progression; B) Cross-sectional area (CSA) of Gastrocnemius muscle. C) Locomotion and exploratory activity D) Sarcopenia classification for control group (n = 7) receiving saline injections and doxorubicin-treated groups (Dox, n = 10) with 6mg/kg every four days (a total dosage of 18 mg/kg). *Indicate significant difference (P<0.05) from control group by student t-test in panels A and C; by ANOVA two-way in panel B, and by Mann-Whitney test in panel B.

Three cycles of chemotherapy treatment with doxorubicin caused reduced static strength (Fig 2A). Static strength reduced significantly (P<0.05) from the tenth to the fifteenth day in the Dox group compared to the control, representing a muscle strength loss of 20% (Fig 2A). Doxorubicin treatment also caused muscle atrophy (-10%) compared to the control group (Fig 2B). The exploratory activity was 33% lower in the Dox group than in the control group (Fig 2C). Upon classification, it was found that 90% of the animals had developed some level of sarcopenia. Specifically, 30% were classified as pre-sarcopenic, 30% as sarcopenic, and 30% as severe sarcopenic (Fig 2D).

### Doxorubicin promotes physical impairments, the hallmark of frailty

**Fig 3:**
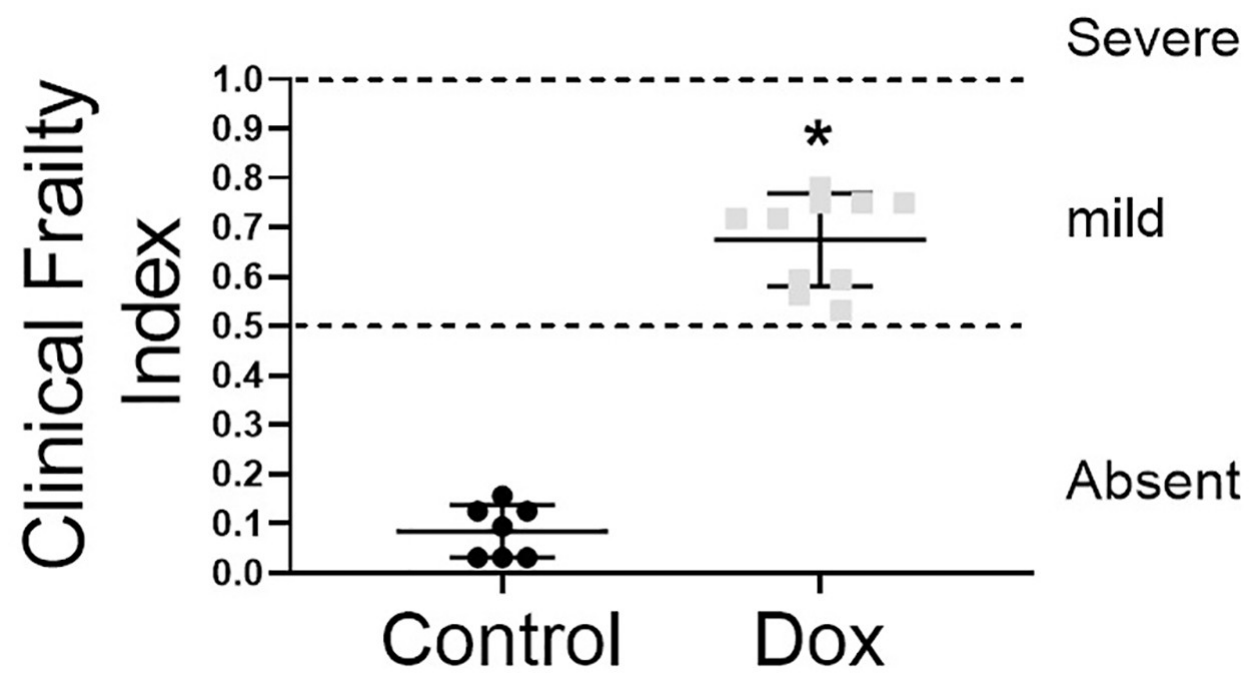
Frailty index and frailty classification for control group (n = 7) receiving saline injections and doxorubicin-treated groups (Dox, n = 10) with 6mg/kg every four days (a total dosage of 18 mg/kg). *Indicate significant difference (P<0.05) from control group by student t-test.

Doxorubicin treatment caused impaired physical function, as demonstrated by significantly (P<0.05) reduced movement duration, traveled maximum and total distance, movement duration, meander, rearing, and traveled velocity compared to the control (Table 1). Thus, doxorubicin treatment significantly increased the frailty score compared to the control (Control: 0.09 x Dox: 0.72). Importantly, all mice in the Dox group presented some level of frailty (Fig 3).

**Table 1:** Eight parameters to calculate frailty.

	Control (n = 7)	Dox (n = 10)	p
Body weight (g)	26.0±2.1	22.5±1.5	0.0021
Movement duration (%)	81.2± 3.2	62.7±9.2	0.0001
Total distance (cm)	3312.5 ± 222,4	2084,31±540.7	0.0001
Maximum distance (cm)	183.5± 54,8	114.6± 25.9	0.0028
Movement duration (s)	243.65 ±9,7	187.95 ± 30.4	0.0001
Meander (degrees/cm)	13± 1.4	24.5±7.8	0.0031
Rearing (events/5 mins)	27,9±10,9	16.3±4.0	0.0001
Velocity (cm/s)	11±0,7	6.9±1.7	0.0001

Values are presented as mean ± SD. Control group receiving saline injections and doxorubicin-treated groups (Dox, n = 10) with 6mg/kg every four days (a total dosage of 18 mg/kg). P< 0.05 indicates significant difference by Student t-test.

## 4 Discussion

The results of our study revealed several important findings: 1) treatment with doxorubicin, administered in cycles, resulted in significant body weight loss, skeletal muscle wasting, strength loss, and impaired physical function. These findings are consistent with the hallmarks of cachexia, sarcopenia, and frailty; 2) The use of doxorubicin-based chemotherapy as a pre-clinical model proved to be valuable in studying cachexia, sarcopenia, and frailty. This model can help further our understanding of these conditions and their underlying mechanisms. 3) We successfully demonstrated that the assessment of body weight loss, skeletal muscle wasting, strength loss, and impaired physical function can be utilized to determine the presence and severity of cachexia, sarcopenia, and frailty in mice. This approach mirrors the methodology used in human studies. Importantly, the association between low muscle mass, decreased strength, impaired physical function, and adverse outcomes in cancer patients has been well-established in the literature [10, 22–25]; however, a pre-clinical model to study these cancer-associated conditions is not well standardized.

### Critique of the experimental approach

Our study addresses a significant gap in the literature by utilizing an experimental model that closely mimics the chemotherapy treatment cycles employed in cancer patients. This is crucial for accurately studying the adverse effects induced by chemotherapy. Unlike most experimental models that use a single high dose of doxorubicin [26], our approach better reflects the treatment regimens administered to patients, allowing for a more realistic evaluation of the effects. Moreover, our study extends beyond traditional assessments of skeletal muscle mass and strength by incorporating functional physical parameters that are assessed *in vivo*. These parameters are often overlooked in experimental research but play a vital role in evaluating the overall impact on physical function. By including these low-cost and easily measurable parameters in our analyses, we provide a comprehensive assessment of the effects of doxorubicin treatment on cachexia, sarcopenia, and frailty. Overall, our study offers valuable insights into the adverse effects of doxorubicin-based chemotherapy and highlights the importance of utilizing relevant and comprehensive experimental models. This knowledge can facilitate the development of effective strategies to mitigate the detrimental effects of chemotherapy and improve the quality of life for cancer patients [27].

The choice of doxorubicin as the chemotherapy approach in our study was based on its widespread use as a first-line treatment for several types of cancer, including lung, breast cancer, and lymphomas [28, 29]. While doxorubicin is effective in killing cancer cells, it is associated with several adverse effects that can significantly impact the quality of life of cancer patients [13]. These include cardiotoxicity, skeletal muscle myopathies, myelosuppression, and fatigue. All of them are associated with muscle wasting and weakness, contributing to the development of cachexia and sarcopenia.

### Chemotherapy treatment with doxorubicin promotes cachexia, sarcopenia, and frailty

Cachexia can have detrimental effects on the overall health and well-being of individuals undergoing chemotherapy. Certain chemotherapy drugs are more strongly associated with the development of cachexia compared to others [30]. Anthracyclines, including doxorubicin, have been suggested to carry a higher risk of inducing or exacerbating cachexia [30, 31]. In fact, our study demonstrated that all mice treated with doxorubicin developed cachexia.

The classification of the animals as pre-cachectic or cachectic based on the observed changes in food intake and body weight used in the present study is in line with the classification used for humans. This classification is clinically relevant as ongoing weight loss is associated with increased chemotherapy toxicity, treatment interruption, and higher mortality rates. By replicating these aspects of cachexia in the animal model, our study provides valuable insights into the impact of doxorubicin treatment on food intake, weight loss, and the development of cachexia. It is important to consider, however, that there are still challenges in studying cachexia. The lack of consensus in defining cachexia leads to discrepancies between studies and hinders the development of strategies to mitigate cachexia [32]. Campelj et al. [33] conducted a study using a chemotherapy regimen with doxorubicin, similar to that used in our study (three doses of 4 mg/kg over 7- day period, totalizing 12 mg/kg), and also concluded that chemotherapy treatment with doxorubicin induces cachexia. However, their analysis was not performed on an individual animal basis, and the food intake was not taken into consideration. Cachexia was determined by the presence of >5% reduction in body, lean, and fat mass, following the classification adapted from Fearon et al. [34].

In addition to the loss of body weight and anorexia, our study also demonstrated that chemotherapy treatment with doxorubicin led to a significant decline in muscle strength, muscle mass, and functional capacity. Indeed, we demonstrated that doxorubicin treatment caused reduced static strength and dynamic strength (S1 Fig). These data indicate the presence of muscle weakness and highlight the negative impact of doxorubicin treatment on muscle strength, which is considered the first hallmark for diagnosing sarcopenia in humans. Previous pre-clinical studies have primarily analyzed specific strength (*ex* vivo) involving acute doses of doxorubicin in various muscle groups, which means that the measurements were conducted on isolated muscle tissue outside of the living organism [26]. For instance, studies have examined specific strength changes *ex vivo* in the diaphragm [35, 36], EDL muscle [37, 38], and soleus muscle [38] after acute doses of doxorubicin. One previous study conducted by Torok, Busekrus, and Hydock [39] explored the strength loss in an *in vivo* model using an acute dose of 15 mg/kg of doxorubicin. They observed a reduction in handgrip strength in rats after 5 days of doxorubicin treatment. Our study extended the existing knowledge by evaluating progressive strength loss over multiple cycles of chemotherapy treatment with doxorubicin in an *in vivo* model.

In humans, loss of muscle mass and physical function are also important components of sarcopenia determination. Our study demonstrated that chemotherapy treatment with doxorubicin resulted in muscle mass loss (reduced cross-muscle weights and cross-sectional area) and impaired physical function. Although some studies have already demonstrated doxorubicin-induced muscle loss [26, 35, 40–44], ours is the first one demonstrating that doxorubicin compromises all three components of sarcopenia–muscle strength, mass, and function. Further research in this area is warranted to expand our understanding of sarcopenia mechanisms in the context of doxorubicin chemotherapy to improve clinical care and physical exercise to mitigate these adverse effects.

In this study, we opted to use the frailty rating protocol proposed by Whitehead *et al*. [21]. Notably, our data revealed that all mice treated with doxorubicin exhibited a mild level of frailty. Our results agree with those found by Lira et al., [45] which determined locomotor functions and sleep patterns in doxorubicin-treated mice (acute dose of 15 mg/kg). These authors demonstrated that doxorubicin-treated mice explored less the environment in which they were placed, presenting a smaller number of crossings in the center of the test area and a smaller number of rearing performed by doxorubicin-treated mice.

It is important to mention that several alternative tests exist to assess physical function in mice, such as the rotarod test, the Morris water maze, and the comprehensive functional assessment battery proposed by Graber [46]. These tests offer complementary measures to evaluate various aspects of sarcopenia and frailty, providing a comprehensive understanding of the functional impairments associated with these conditions in mice. However, it is worth noting that these tests often require multiple pieces of equipment, including a treadmill and/or a rotarod apparatus, which can be expensive and may not be available in all laboratories. In contrast, the test proposed in our study only requires one camera, open-source software, and an arena, making it a cost-effective alternative that can be more accessible for research purposes.

### Strengths and limitations

Despite all the strengths mentioned in the discussion, this study presents some limitations that should be mentioned. First, it should be noted that our study did not account for potential differences between sexes, and therefore, the findings may not be directly applicable to both males and females. Given the known physiological and hormonal variations between genders, further research is needed to investigate the impact of doxorubicin treatment and its association with cachexia, sarcopenia, and frailty in a sex-specific manner. Secondly, it is important to note that this study did not include a cancer model for treatment with doxorubicin. Therefore, the findings may not fully capture the complex interactions between doxorubicin, cancer, and the development of cachexia, sarcopenia, and frailty. These limitations emphasize the need for further pre-clinical models that incorporate the cancer aspect to better understand the adverse effects of doxorubicin on these conditions.

## 5 Conclusion

Our study successfully demonstrated that the administration of doxorubicin in cycles closely mimics the human chemotherapy model and induces a range of detrimental effects such as body weight loss, muscle wasting, loss of strength, and physical impairments in mice. These observed changes allowed for the identification of the presence and severity of doxorubicin-induced cachexia, sarcopenia, and frailty in this animal model. The ability to replicate and assess these conditions in a pre-clinical model is crucial for understanding the mechanisms underlying cachexia, sarcopenia, and frailty induced by doxorubicin treatment. It provides valuable insights into the potential adverse effects experienced by cancer patients undergoing doxorubicin chemotherapy and contributes to the development of effective interventions to mitigate these effects.

## Supporting information

S1 Fig Hallmarks of sarcopenia and sarcopenia classification. (TIF)

S1 Data (DOCX)
