# Supplementary figures and images for: Doxorubicin causes cachexia, sarcopenia, and frailty characteristics in mice
Source: PLoS One. 2024 Apr 22;19(4):e0301379. doi: 10.1371/journal.pone.0301379 (PMC11034664; doi:10.1371/journal.pone.0301379)

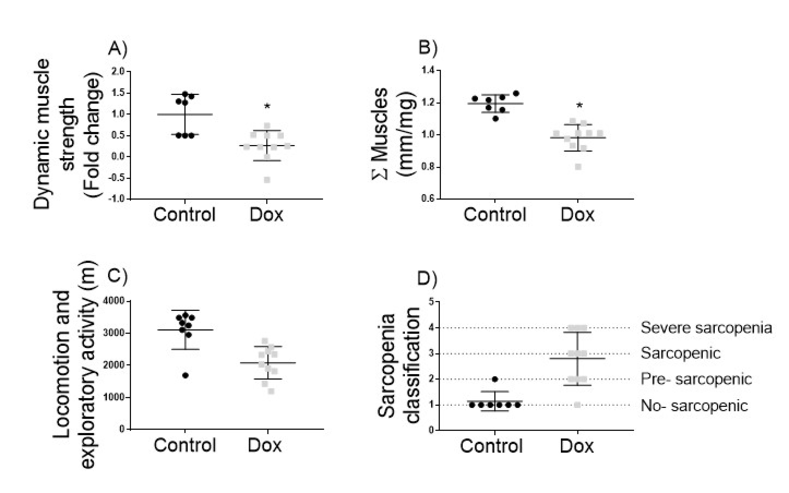

Supplement: S1 Fig — (TIF) [file pone.0301379.s001.tif]
