# Supplementary material for: Doxorubicin causes cachexia, sarcopenia, and frailty characteristics in mice
Source: PLoS One. 2024 Apr 22;19(4):e0301379. doi: 10.1371/journal.pone.0301379 (PMC11034664; doi:10.1371/journal.pone.0301379)
Supplement: S1 Data — (DOCX) [file pone.0301379.s002.docx]

**Supplementary data**

**Methods**

*Alternative muscle strength and mass loss for sarcopenia determination*

To provide an alternative measure of muscle mass, we summing up the mass of skeletal muscles, including the gastrocnemius, soleus, plantar, extensor digitorum longus, and tibialis anterior [16]. The tibia bone was dissected, and its length was measured. The sum of skeletal muscle mass was then adjusted by the tibia bone length.

With the same purpose, we conducted an alternative measure of strength, we conducted a ladder climbing test, which assesses dynamic strength. The ladder climbing apparatus was adapted to accommodate mice (a length of 0.55m and a grid spacing of 0.7cm- the incline of the laffer was set at 80°). This setup allowed mice to performed 8–12 dynamic movements per climb. This method was previously describe in studies by Padilha et al., 2019 [46]. and Testa et al., 2022 [47]. At the top of the ladder, a dark covered chamber was constructed for interval resting between climbing bouts. The ladder climbing test was conducted with loads attached to their tails.

Before the administration of the drug, the mice underwent a period of adaptation without any load for 5 days. In the first test (pre-treatment), the initial load was set at 100% of their body mass. The mice were allowed to perform four to eight ladder climbs with a two- minute rest period between each attempt. If the mice failed to complete the climb, the load was maintained, and the mice was given one more attempt. The maximum load for each mouse was determined, after experiencing up to two consecutive failures with the same load. For every successful attempt, the load was increased by 10g in the first two attempts and by 5g from the third attempt onwards until the mice was no longer able to climb.

The second test (post-treatment) was performed 48 hours before the euthanasia. In this test, the initial load was set at 80% of the maximum load achieved in the first test. The load adjustment was made by adding or subtracting 5g from the mice’s load weight. If the mice failed to complete the climb after two attempts, the load was decreased by 5g until the mouse was able to successfully complete the climb. Likewise, If the mice successfully completed the climb, the next load was set to 100% of their body mass, and then the load was increased by 5g for each subsequent attempt.

**Results**

Dox group had a poor performance (p<0.05) compared to control group in the dynamic strength test. The average reported load from control group was 19g and Dox group loaded 5.2g, representing that dox group carried a load 72% lighter than the load carried by control (supplementary figure 1B). Doxorubicin treatment also caused reduced muscle mass (-18%). Using these parameters, 90% had developed some level of sarcopenia; 20% were classified as pre-sarcopenic, 20% as sarcopenic and 50% as severe sarcopenic (supplementary figure 1D).

**Supplementary figure 1.** Hallmarks of sarcopenia and sarcopenia classification. A) Dinamic muscle strength; B) Sum of muscle. C) Exploratory activity D) Sarcopenia classification for control group (n=7) receiving saline injections and doxorubicin-treated groups (Dox, n=10) with 6mg/kg every four days (a total dosage of 18 mg/kg). *Indicate significant difference (P<0.05) from control group by student t-test in panels A and C; by ANOVA two-way in panel B, and by Mann-Whitney test in panel B.

|  | |  |  |  | |  |  |  |  |  |  |
| --- | --- | --- | --- | --- | --- | --- | --- | --- | --- | --- | --- |
|  | **Supplementary table 1.** Incidence of sarcopenia with different methods of measuring strength and muscle mass in animals. | | | | | | | | | | |
| Sarcopenia parameters | | Static strength+ CSA+ Loc. | |  | Static strength+  Σ+ Loc. | | | Dinamic strength +CSA+ Loc. | | Dinamic strength+ Σ+ Loc. | |
|  |  | Control | Dox | Control | |  | Dox | Control | Dox | Control | Dox |
| non-sarcopenic | | 6 (86%) | 1 (10%) | 6 (86%) | |  | 1 (10%) | 5 (71%) | 1 (10%) | 5 (71%) | 1 (10%) |
| pre-sarcopenic | | 0 | 3 (30%) | 1 (14%) | |  | 2 (20%) | 1(14) | 2 (20%) | 2 (29%) | 2 (20%) |
| Sarcopenic | | 1 (14%) | 3 (30%) | 0 | |  | 2 (20%) | 1(14) | 3 (30%) | 0 | 2 (20%) |
| severe sarcopenia | | 0 | 3 (30%) | 0 | |  | 5 (50%) | 0 | 4 (40%) | 0 | 5 (50%) |
|  | |  |  |  | |  |  |  |  |  |  |

Data are presented in absolute (relative) values from control group (n=7) receiving saline injections and doxorubicin-treated groups (Dox, n=10) with 6mg/kg every four days (a total dosage of 18 mg/kg).
